# A program evaluation of an in-school daily physical activity initiative for children and youth

**DOI:** 10.1186/s12889-018-5943-2

**Published:** 2018-08-16

**Authors:** Emily Bremer, Jeffrey D. Graham, Scott Veldhuizen, John Cairney

**Affiliations:** 1 Department of Kinesiology, McMaster University, Hamilton, Canada; 2 INfant and Child Health (INCH) Lab, Department of Family Medicine, McMaster University, Hamilton, Canada; 3 Faculty of Kinesiology and Physical Education, University of Toronto, WSB, 55 Harbord Street, Toronto, ON M5S 2W6 Canada

**Keywords:** Intervention, Health policy, Psychosocial health, Education

## Abstract

**Background:**

The school system is one setting in which children’s physical activity levels may be increased through daily physical activity (DPA) policies and initiatives. Adherence to DPA policies is typically poor and results are limited in regard to the associated benefits for participating children. Therefore, the purpose of this study was to evaluate a range of psychosocial outcomes following a community-led, in-school DPA initiative for 9–14 year old children and youth.

**Methods:**

This program evaluation examined the impact of a DPA program consisting of 20 min of teacher-led DPA for 20 consecutive weeks. Student outcomes were measured using a questionnaire administered at three time points: baseline, mid-intervention, and post-intervention. A teacher questionnaire regarding program adherence and student behaviour was completed at post-intervention. Mixed effects models were used to test for intervention effects, with random intercepts for students, classes, and schools, as well as fixed effects for age and sex. Due to the large number of outcomes measured, we first conducted an omnibus test of the intervention effect followed by three exploratory analyses examining each outcome separately, associations between outcomes and program adherence, and results from the teacher survey.

**Results:**

Thirty classes (*N* = 19 experimental) from 7 schools participated in the study, with a total of 362 children (*n* = 265 experimental). There was no significant overall effect of the intervention (z = 0.89, *p* = 0.38) and the exploratory analyses demonstrated significant differences only for self-esteem and subjective happiness, with the control group slightly increasing relative to the experimental group. Teacher-reported adherence to the program was poor with only 21% of teachers adhering to the program. There was no association between overall adherence and student reported outcomes; however, positive correlations were present between adherence and teacher-reported student behaviour.

**Conclusions:**

The DPA program evaluated here did not improve the psychosocial well-being of elementary school-aged children more than usual practices. However, adherence to the program was poor and it did not have a negative effect on the students. Future work is needed on how best to support DPA implementation in the context of the school day and how student well-being may be positively impacted through school-based physical activity.

**Trial registration:**

Program Evaluation of an in-school Daily Physical Activity Initiative NCT03618927, August 6, 2018. Retrospectively registered.

## Background

There is considerable evidence regarding the impact of daily physical activity (DPA) on youth development and health [1, 2]. Children and youth who engage in higher levels of physical activity are more physically fit [3] and coordinated [4], exhibit improved executive functioning [5–7], and perform better academically [8, 9] when compared to children who are less active. Research has also demonstrated positive associations between physical activity and psychosocial outcomes including self-efficacy [10, 11], self-concept [11–13], and social support [14]; and lower levels of depression, internalizing, and externalizing symptoms [12]. Further, it has been proposed that psychosocial factors such as self-perception and social connectedness may mediate the relationship between physical activity and global mental health and well-being [2]. Despite the positive benefits of activity, 91% of Canadian children and youth are not engaging in the recommended 60 min of daily moderate to vigorous physical activity (MVPA) [15, 16].

One way in which we may positively influence the physical activity patterns of children and youth is through the school system, as a large portion of each child’s day is spent in that setting. The evidence, however, is mixed in regard to the benefits of school-based physical activity interventions [17, 18]. For example, evidence supports the benefit of in-school physical activity programs for improvements in academic performance [17, 18]. However, it would appear that school-based physical activity programs have little to no effect on physical activity levels outside of the program or on health indicators such as physical fitness [18].

Most of this evidence stems from programs designed and implemented by research teams rather than program evaluation (and collaboration) of new or existing practices within the school [19]. Researcher-led programs typically do not require a high degree of buy-in for implementation (beyond gaining access initially) from school teachers and administrators, as they are led by the research team or involve considerable support for training and incentives. Once researchers leave, these supports are often lost. It is therefore essential to evaluate programs developed within the educational system, as these were designed for long-term use, and are appropriately resourced given specific contexts.

In Ontario, Canada, there has been an attempt to increase school-day physical activity through a 2005 DPA Policy released by the Ministry of Education [20]. The policy mandates that all publicly funded elementary schools (Grades 1–8) provide a minimum of twenty minutes of sustained MVPA each school day during instructional time [20, 21]. Despite this promising mandate, adherence to the policy has been poor [22–24]. For example, Stone et al. [24] objectively assessed the physical activity levels of a large sample of Ontario children in the context of their school day and classroom schedules. They found that less than 50% of the participating children were provided with DPA and that none engaged in 20 min of sustained MVPA, with most activity bouts lasting 5–10 min [24]. More recently, Allison et al. [22] surveyed school administrators and classroom teachers from across Ontario regarding fidelity to the implementation of the DPA policy. They found that 61.4% of administrators reported meeting DPA policy requirements at the school level; yet only 50% of classroom teachers reported the requirements being met on a classroom level. These results align with the objectively measured results of Stone et al.’s [24] research and together suggest that adherence to the policy is poor.

The recent evaluation of Ontario school teachers and administrators also identified a number of factors related to adherence to the DPA policy [22]. They found that teachers who felt more confident about planning and implementing DPA were more likely to report having done so successfully; however, only 60% of teachers expressed confidence in these areas. Further, only 15% of teachers reported often or always using DPA resources and supports, yet using resources was consistently related to implementation fidelity [22]. These findings underscore the need to improve teacher confidence as well as to directly guide and support implementation.

Given low rates of compliance, some school boards in Ontario have partnered with external organizations and/or have developed in-school programming to improve compliance with the policy. Our involvement in this study was on behalf of an organization who has been working with local schools proximate to our university to increase DPA using a structured, teacher-led program of in-class exercises. Given the literature on the impacts of physical activity to psychosocial outcomes in children and youth [10–14], both the community organization and the partnering schools were interested in testing if participation in the program led to improvements in psychosocial outcomes. Our research team designed and implemented an evaluation project to examine these outcomes in this context. Specifically, the purpose of this program evaluation was to examine the impact of a community-led, in-school DPA program on a range of psychosocial outcomes including, self-efficacy, self-regulation, emotional well-being, school belonging, and grit in 9–14 year old children and youth, in comparison to children not participating in the program. Secondary outcomes included teacher-reported adherence to the program and the influence of adherence on the primary outcomes.

## Method

### Intervention

The intervention consisted of a DPA program designed by a national organization with expertise in school-based physical activity programming and delivered in school by teachers. The program was offered to students in grades 4 through 8 and consisted of 20 min of structured DPA in school for 20 consecutive weeks. The DPA activities included jumping jacks, squats, running and other body weight exercises. School teachers and student leaders attended a one-day workshop on how to deliver the program as part of regular school activities and were provided with instructional materials to take back to their school for program delivery. This workshop was intended to increase teachers’ confidence to implement DPA through the use of the manual and supporting resources (e.g., video modules, workbooks, etc.). At the conclusion of the 20 weeks, participating students were invited to participate in a 5 km fun run/walk with students and teachers from participating schools, and community leaders from within the geographical region in which the program took place. Schools and teachers were encouraged to approach the program as a “team,” emphasizing pro-social aspects of team work and collective impact on behavioural (individual) and social change. This was reinforced at the annual fun run/walk where schools run together as a group, along with children from across participating schools. Moreover, the organization promotes the idea of physical activity and participation to both teachers and students as means to not only improve physical health, but also mental and social well-being. Given the developmental benefits associated with engagement in regular physical activity, and the positive developmental environment that the program strives to achieve, it is possible this program had the potential to positively impact children’s social and psychological well-being through increased DPA.

### Participants and design

Seven schools from a local Catholic school board were chosen by the school board to participate in this program evaluation. Teachers were asked to participate by their school administrators. Between one and seven classes participated in each school, for a total of 30 participating classes. All classes were between grade 4 and grade 8 (see Table 1 for the number of participating classes in each grade), with participants between 9 and 14 years old (*M*_*age*_ = 11.7y, *SD* = 1.3y, *n* = 176 females). All students (*n* = 783) in the participating classes were approached to participate in the study. We received written parental consent and child assent from 362 students (46.2%). Teachers had previously chosen whether or not their class would participate in the program or to act as a control class, and this determined the final groups. This program evaluation study therefore included 19 classes and 265 students participating in the DPA program, and 11 classes and 97 students participating in their typical classroom routines. Only teachers who had chosen to participate in the program received the DPA program training. The remaining teachers were however still expected to provide DPA to their students, as per the Ontario education curriculum [20]. Table 2 provides an overview of participating classes and students by school. The study was approved by an Institutional Research Ethics board and the local school board. This trial was retrospectively registered with ClinicalTrials.gov on August 6, 2018 as Program Evaluation of an in-school Daily Physical Activity Initiative NCT03618927.

**Table 1:** Number of classes in each grade by group

Number of classes		
Grade	Experimental	Control
4	3	1
4/5	2	2
5	2	0
5/6	2	1
6	2	4
7	3	1
7/8	1	1
8	4	1

**Table 2:** Number of participating classes and participants by school

Number	School 1	School 2	School 3	School 4	School 5	School 6	School 7
Control classes (N)	0	0	0	2	6	3	0
Experimental classes (N)	5	1	3	4	1	3	2
Total classes (N)	5	1	3	6	7	6	2
Control participants (n)	0	0	0	16	58	23	0
Experimental participants (n)	74	24	59	31	18	37	22
Total participants (n)	74	24	59	47	76	60	22

*N* Number of classes, *n* number of participants

### Procedure

Outcomes were measured using a questionnaire administered at three time points: baseline, mid-intervention (10 weeks), and post-intervention (within 2 weeks post-intervention). Teachers of classes receiving the intervention completed a brief questionnaire post-intervention to assess fidelity to the intervention, while teachers of control classes completed a questionnaire about usual classroom routines.

All questionnaires were administered in class using Android NeuTab Pro™ tablets provided by the research team. Participants who preferred to complete the questionnaire on paper were able to do so. The student questionnaire took approximately 30 min to complete, was composed of subscales drawn from existing questionnaires, and was identical at each time point, with the exception of demographic questions, which were asked only at baseline. The teacher questionnaire took approximately 10–15 min to complete. Research assistants were on hand during administration to assist with completion and answer any questions students may have had. Students and teachers were provided with a small incentive ($5 and $10 gift cards for students and teachers, respectively) for their time in the study.

### Measures

#### Student questionnaire

A 92-item questionnaire was developed for this study. It assessed demographics, aspects of psychosocial well-being, well-being associated with school, and engagement in physical activity in school and outside of school. Scales and subscales were drawn from existing validated measures.

##### Demographic information

Participants were asked for their birth date, gender, ethnicity/cultural heritage, and postal code, as well as about the composition of their household.

##### Mastery

Mastery which refers to “*the extent to which people see themselves as being in control of the forces that importantly affect their lives,”* [25] was assessed with the Pearlin Mastery Scale [25]. Pearlin’s Mastery Scale includes seven-items scored on a four-point Likert-type scale ranging from 1 (*Strongly Disagree*) to 4 *(Strongly Agree*). An example item is “*I can do just about anything I really set my mind to.*” One of the seven items (“*What happens to me in the future mostly depends on me*”) was excluded for this study, and the first item was modified from the negative to the positive to ease comprehension for younger children. The average internal consistency of the measure across the three time points was Cronbach’s *α* = 0.70.

##### Self-regulation

Self-regulation was assessed using a subset of 7 items drawn from the Adolescent Self-Regulatory Inventory [26], a 36-item self-report measure designed to assess self-regulation in late childhood and adolescence. The measure consists of two scales, one that measures short-term regulation (13 items) and one that measures long-term regulation (14 items). A subset of 7-items was selected for the measure, most related to short-term self-regulation (i.e., 5-items from the short-term self-regulation subscale). An example item from the short-term self-regulation scale is: *“When I’m sad, I can usually start doing something that will make me feel better.”* An example item from the long-term self-regulation scale is: *“If I really want something, I have to have it right away.”* Items are scored on a five-point Likert-type scale ranging from 1 (*Not at all true for me*) to 5 (*Really true for me*). The scale has demonstrated concurrent validity, being positively associated with pro-social behaviour and negatively associated with externalizing and internalizing problems [26]. The average internal consistency of the measure was *α* = 0.61.

##### Emotional well-being, behavioural difficulties, and pro-social behaviour

Emotional well-being, behavioural difficulties, and pro-social behaviour were measured using the self-report version of the Strengths and Difficulties Questionnaire (SDQ) [27, 28]. The SDQ is a widely-used brief assessment of emotional well-being, behavioural difficulties, and pro-social behaviour among children and adolescents (e.g., [29, 30]). It includes 25 items across 5 subscales (hyperactivity, emotional symptoms, conduct problems, peer problems, and pro-social) scored on a three-point Likert-type scale ranging from 1 (*Not True*) to 3 *(Certainly True*). An example item is “*I get a lot of headaches, stomach-aches, or sickness.*” The self-report SDQ has demonstrated good psychometric properties with diverse groups of children and adolescents [29–31]. The average internal consistency of the measure was *α* = 0.80.

##### Self-esteem

Self-esteem was measured using the Rosenberg Self-Esteem Scale [32], a 10-item measure that has shown to have good construct validity in children [33]. The scale is scored on a four-point Likert-type scale ranging from 0 (*Strongly Disagree*) to 3 (*Strongly Agree*). An example item is “*On the whole, I am satisfied with myself*.” The measure’s average internal consistency was *α* = 0.86 across the three time points.

##### Grit

Grit, defined as *“perseverance and passion for long-term goals,*” [34] was assessed with the 8-item Short Grit Scale for Children [34, 35]. The items are scored on a five-point Likert-type scale ranging from 1 (*Not at all like me*) to 5 (*Very much like me*). An example item is *“New ideas and projects sometimes distract me from previous ones*.” The average internal consistency of the measure was *α* = 0.71.

##### Global happiness

Global happiness was measured using the 4-item Subjective Happiness Scale [36]. The scale is scored on a seven-point Likert-type scale. Each item has its own stem and anchors ranging from 1 to 7. An example item stem is *“In general I consider myself …*” with 1 anchored as *Not a very happy person* and 7 anchored as *A very happy person.* The scale has demonstrated good psychometric properties in children and adolescents [36–38]. The average internal consistency of the measure was *α* = 0.74 across the three time points.

##### Commitment to school

Commitment to school was assessed with 5 items drawn from the 10-item Commitment to School scale [39]. The scale consists of 10-items scored on a four-point Likert-type scale ranging from 1 (*Strongly Disagree*) to 4 (*Strongly Agree*). A subset of five-items was selected, scored on a five-point Likert-type ranging from 1 (*Not at all true for me*) to 5 (*Really true for me*). An example item is “*You try really hard at school*.” The average internal consistency of the measure was *α* = 0.80.

##### Sense of belonging at school

Sense of belonging at school was measured using the 5-item short form version of the Sense of Belonging Scale [40]. An example item is “*I feel comfortable at my school.*” The measure’s average internal consistency was *α* = 0.86.

##### Physical activity

Physical activity was assessed with the Physical Activity Questionnaire for Older Children (PAQ-C) [41]. The PAQ-C was designed for children in grades 4–8 who have recess as a regular part of their school week. The PAQ-C is a 9-item 7-day recall instrument that has been shown to have good psychometric properties [42]. We omitted the first item from this questionnaire because it asked about the types of physical activity performed, and we were interested only in overall levels of participation. An example item is “*In the last 7 days, what did you do most of the time at recess?*” The average internal consistency of the measure was *α* = 0.82.

##### Task self-efficacy

Self-efficacy for engaging in physical activity, sports, and active play was assessed using a three-item scale adhering to recommendations by Bandura [43, 44] for assessing self-efficacy. Each item was prefaced with the stem “*I am confident in my ability to engage in …*” The individual items were “*Physical activity (e.g., running, yoga, skating),*” “*Sports (e.g., soccer, baseball, ultimate Frisbee),*” and “*Activity play (e.g., playing with friends at recess or after school).*” Following guidelines provided by Bandura [43, 44], participants rated their confidence for each item using an 11-point scale (0 *= not confident,* 10 = *totally confident)*. A generalized task self-efficacy overall score was computed by averaging the ratings for each item to produce a scale value out of 10. The measure’s average internal consistency was *α* = 0.86.

#### Teacher questionnaire

A 21-item questionnaire was developed for this study. Completed by the homeroom teacher at the last measurement point, it included 3 sections: adherence to the program, student behaviour, and physical activity opportunities. Questions regarding adherence to the program were completed by the experimental classroom teachers only and included questions such as, *“On average, how many days per week did your students participate in the program?”* Questions regarding student behaviour were completed by all participating teachers and included behavioural ratings of student behaviour (e.g., *“To what extent did you see changes in your student’s behaviour between January and now [in self-confidence, attentional control, academic performance, emotional regulation, self-control, and social interactions]*?” on a 10-point scale from “*No improvement*” to “*A great deal of improvement.*” Lastly, questions pertaining to student engagement in school-based physical activity (i.e., number and length of physical education classes and recesses per week) were completed by all participating teachers.

### Data analysis

We first produced descriptive statistics and tested for overall group differences using t-tests (for continuous variables), chi-square tests (for nominal ones), and a Wilcoxon test (for number of siblings).

To test for intervention effects, we used mixed effects models. Participants were assessed on three occasions, and the intervention was administered at the class level. We therefore included random intercepts for students, classes, and schools, as well as fixed effects for age and sex. To test for intervention effects, we included a group*time interaction in each model.

Given the large number of outcomes, we conducted an omnibus test of the intervention effect using the approach suggested by O’Brien [45], which involves first ranking participants on each outcome and then using the sum of these ranks as the primary outcome. We also conducted three exploratory analyses. First, we examined each outcome separately. Second, as fidelity to the program was expected to vary, we tested for associations within the intervention group between outcomes and the amount of exercise actually performed (obtained from the teacher survey). Finally, we explored results from the teacher survey for changes in perceived student behaviour and opportunities for participation in physical activity, as well as for the associations between adherence and perceived changes in student behaviour. All analyses were conducted in Stata 14 [46].

## Results

The final sample included 362 children with consent. Of these, 265 received the intervention, while 97 served as controls. Attrition by the study end was 16% in the intervention group and 20% among the controls (*X*^*2*^ = 0.57, df = 1, *p* = 0.45). The characteristics of the sample at baseline are shown in Table 3.

**Table 3:** Descriptive characteristics of the sample at baseline

	Control	Experimental	Difference
Age (mean (SD))	11.6 (1.2)	11.7 (1.3)	*t* = 0.27,df = 360,*p* = 0.79
Female	48 (49%)	128 (48%)	*X*^*2*^(1) = 0.04,*p* = 0.84
Male or other	49 (51%)	137 (52%)	
White	35 (36%)	124 (47%)	*X*^*2*^(1) = 3.31,*p* = 0.07
Other	62 (64%)	141 (53%)	
Lives in one-home	15 (17%)	41 (17%)	*X*^*2*^(1) = 0,*p* = 0.96
Splits time between two-homes	73 (83%)	203 (83%)	
Two-parent home	29 (30%)	71 (27%)	*X*^*2*^(1) = 0.34,*p* = 0.56
Other	68 (70%)	194 (73%)	
Number of siblings	1.5 (1.1)	1.3 (1.1)	z = 1.63,*p* = 0.11
Both parents born in Canada	12 (17%)	22 (15%)	*X*^*2*^(1) = 0.19,*p* = 0.67
One parent born elsewhere	58 (83%)	126 (85%)	

Our omnibus test of the intervention showed no significant overall effect (z = 0.89, *p* = 0.38). Results of the exploratory analysis of individual outcomes (Table 4) showed significant differences only for self-esteem and subjective happiness. Self-esteem increased slightly in the intervention group, but by less than in the control group, while subjective happiness improved somewhat among controls but remained essentially the same over time in the intervention group (Table 5). These and all other observed intervention effects were small, with standardized effects sizes (Cohen’s *d*; [47]) below 0.15.

**Table 4:** Model results for individual outcomes

Outcome	Group*time effect (95% CI)	p
Mastery	−0.2 (− 0.52–0.12)	0.23
Self-regulation	0.14 (−0.32–0.59)	0.56
SDQ Emotional problems	0.11 (−0.14–0.36)	0.38
SDQ Conduct problems	−0.03 (− 0.2–0.14)	0.72
SDQ Hyperactivity	−0.09 (− 0.31–0.13)	0.41
SDQ Peer problems	0.07 (− 0.12–0.26)	0.45
SDQ Prosocial	−0.13 (− 0.35–0.09)	0.25
SDQ Total score	0.04 (− 0.44–0.51)	0.88
Self-esteem	−0.52 (− 0.97 - -0.07)	**0.02**
Grit	−0.57 (−1.19–0.04)	0.07
Subjective happiness	−0.6 (−1.07 - -0.14)	**0.01**
Commitment to school	−0.23 (− 0.52–0.07)	0.14
Sense of belonging	−0.18 (− 0.57–0.21)	0.36
Physical activity	− 0.07 (− 0.16–0.01)	0.09
Generalized task self-efficacy	0.1 (− 0.5–0.69)	0.76
Physical activity self-efficacy	0.04 (− 0.23–0.3)	0.79
Sports self-efficacy	0.01 (−0.24–0.27)	0.92
Active play self-efficacy	0.03 (−0.22–0.28)	0.82

*SDQ* Strengths and Difficulties Questionnaire

*indicates an interaction (i.e., Group by time effect)

bold data indicates the value is significant at *p*<.05

**Table 5:** Group by time results for individual outcomes

Outcome	Control group (*n* = 72–97)			Intervention group (*n* = 201–260)		
	Time 1 Mean (SD)	Time 2 Mean (SD)	Time 3 Mean (SD)	Time 1 Mean (SD)	Time 2 Mean (SD)	Time 3 Mean (SD)
Mastery	17.4 (3.1)	18.3 (3.5)	18.3 (3.6)	17.5 (2.9)	17.8 (2.9)	18.2 (3)
Self-regulation	17 (4.4)	17.5 (4.3)	17.5 (4.5)	16.7 (4.6)	17.4 (4.8)	17.5 (4.4)
SDQ Emotional problems	3.2 (2.5)	2.8 (2.7)	2.6 (2.6)	3.3 (2.6)	3.1 (2.5)	3 (2.5)
SDQ Conduct problems	1.7 (1.7)	1.8 (1.7)	1.6 (1.8)	1.7 (1.6)	1.6 (1.5)	1.6 (1.7)
SDQ Hyperactivity	3.1 (2.1)	3.2 (2.3)	3.1 (2.3)	3.4 (2.3)	3.3 (2.3)	3.3 (2.3)
SDQ Peer problems	1.9 (1.7)	1.7 (1.8)	1.9 (1.9)	2.1 (1.7)	2.1 (1.9)	2.2 (1.9)
SDQ Prosocial	8.6 (1.8)	8.7 (1.6)	8.9 (1.6)	8.5 (1.6)	8.5 (1.8)	8.3 (1.8)
SDQ Total score	9.9 (5.7)	9.4 (6.1)	9.2 (6.5)	10.5 (6)	10 (6)	10 (6.2)
Self-esteem	30.8 (6)	32.6 (5.8)	32.2 (6.1)	31.1 (5)	31.5 (5.3)	31.8 (5.6)
Grit	20.3 (4.3)	21.3 (3.9)	21.9 (4.6)	20.8 (3.8)	21.6 (4.1)	21.2 (4.8)
Subjective happiness	21.5 (4.8)	21.5 (5.1)	22.3 (4.8)	21.5 (4.4)	21.3 (4.4)	21.4 (4.2)
Commitment to school	21.2 (3.7)	21.2 (3.7)	21.6 (3.5)	21.4 (3.2)	21.4 (3.1)	21.6 (3.2)
Sense of belonging	20.1 (4.3)	20 (4.5)	20.1 (4.9)	20.4 (3.7)	20.2 (3.7)	20.4 (3.8)
Physical activity	3.1 (0.8)	3.3 (0.8)	3.4 (0.7)	3.2 (0.9)	3.3 (0.8)	3.5 (0.9)
Generalized task self-efficacy	8.1 (1.7)	8.3 (1.9)	8.2 (2.0)	8.0 (2.0)	8.1 (2)	8.1 (2.1)
Physical activity self-efficacy	7.9 (2)	8.2 (1.9)	8.1 (2.2)	7.7 (2.4)	7.9 (2.3)	8 (2.3)
Sports self-efficacy	7.9 (2.4)	8.1 (2.4)	8 (2.4)	7.9 (2.4)	7.9 (2.4)	7.9 (2.4)
Active play self-efficacy	8.7 (1.9)	8.7 (1.9)	8.6 (2.1)	8.4 (2.1)	8.4 (2.1)	8.3 (2.2)

*SDQ* Strengths and Difficulties Questionnaire

Within the experimental group, teachers reported engaging in 30 to 100 min of exercise per week, with only 4 experimental classes (4/19, 21%) adhering to the mandated 100 min. The amount of exercise performed in the experimental classes (according to teacher report) during the program averaged 70.7 (SD = 23.0) minutes per week and was not associated either with our omnibus outcome (z = 0.64, *p* = 0.52) or with any of the individual outcome variables. There were no significant differences in the quantity of physical education classes in the experimental and control classes over the intervention period (t(27) = − 0.23, *p* = 0.82). Further, all control classes also reported providing their students with opportunities to be physically active throughout the school day, beyond physical education and recess, in order to align with curricular mandates. The teachers in the control classes reported that these opportunities mainly consisted of 10–20 min of DPA (in the form of short games, dance videos, and fitness videos) completed on days when physical education classes did not take place.

In the experimental group, teachers reported a relatively high level of self-efficacy (mean = 7.3 ± 1.9 out of 10) in their ability to lead the program, and positive correlations between adherence to the program and teacher reported perceptions of change in student behaviours were present. More specifically, significant correlations were evident between program adherence and teacher-reported student attentional control (*r* = .48, *p* < .05), emotional regulation (*r* = .46, p < .05), and social interactions (*r* = .60, *p* < .01).

## Discussion

Mandatory DPA has now been in policy for over a decade in Ontario, yet there is strong evidence to indicate a lack of adherence to this policy [22–24]. While we know that regular participation in physical activity has numerous psychosocial benefits for children and youth [10–12, 14], it is not well-understood if school-based physical activity programs result in similar outcomes. The program evaluated in this study is one way in which DPA implementation may be supported in schools through the provision of training and resources for classroom teachers. The effectiveness of the program at improving psychosocial outcomes and adherence had previously not been examined but, was of interest to the national organization who designed the program given current evidence regarding DPA adherence [22] and the associations between physical activity and psychosocial well-being [10–12, 14].

Results indicate that this program did not have any significant overall effect on the psychosocial well-being of participants. Results of the exploratory analysis, moreover, showed small but significant negative effects on self-esteem and subjective happiness. These results do not show harm, as children in both groups improved overall; as the experimental group improved less, however, they may imply that the intervention was less effective than the activities it displaced, or that it hindered some other process of improvement. The interpretation of these results is, however, complicated. They arise from an experimental analysis uncorrected for multiplicity; the nature of the study, which was quasi-experimental, cannot exclude selection effects, or differential attrition related to unmeasured variables.

There are a few possible reasons for these findings. First, adherence was poor, with only 4 (21%) teachers reporting daily adherence and most only engaging in the program 3–4 days per week. The low rate of adherence is alarming as 20 min of DPA is the standard that should be implemented by all classroom teachers [20]. That providing a structured program such as this still does not improve adherence is concerning. Interestingly, we did find positive correlations between program adherence and teacher-reported behavioural outcomes, suggesting that there may be opportunity for improvements in student outcomes if adherence is improved.

Although 20 min of DPA is the expected standard in Ontario [20], even a structured program with teacher supports did not consistently meet this target. This is consistent with previous research investigating adherence to the Ontario DPA policy [22], in addition to adherence to researcher-led school-based physical activity interventions. For example, Donnelly et al. [48] implemented a comprehensive 3-year school-based physical activity intervention in which teachers were to deliver 20 min of physically active lessons 5 days per week over a 3-year period. Teacher-reported adherence, however, indicated that these lessons were only delivered 3 days per week, for an average of 55 min of activity per week, across the intervention period [48].

Poor adherence to these interventions may be due to the competing interests and pressures facing elementary school teachers. Allison et al. [22] reported that over 75% of teachers in their study agreed or strongly agreed that competing curricular priorities and a lack of time were barriers to DPA implementation. Although teachers in the current study reported a relatively high degree of self-efficacy to implement the program, it is plausible that they did not see it as a priority in the context of the greater school curriculum. This is clearly an important barrier to both research and practice. Programs initiated by outside organizations such as the one examined here, in particular, may need to offer ongoing support and reminders, as there is often little contact with teachers following the initial training sessions. Additionally, supporting teachers and administrators in conducting on-going monitoring and self-evaluations of program implementation and student outcomes may also provide a sense of ownership over DPA programs to improve adherence.

One means to achieve these goals is through professional development activities for teachers. According to the literature on best practices in this area, in addition to the aforementioned considerations, other strategies should include active learning strategies for teachers (e.g., accomplished by provision of engagement opportunities and by tracking teachers behaviours), by making DPA coherent with student learning (e.g., record and monitor teacher-derived goals; connect activities to focus on student achievement) and by building a community of practice within school boards on physical activity (e.g., provide teachers with release time to fully engage with the material) [49]. Indeed, the Comprehensive School Physical Activity Program (CSPAP; [50, 51]) is one approach that includes several on-going training strategies for training teachers and staff to incorporate DPA into the school day (also see [19] for an overview). Thus, further research testing the effect of professional developmental strategies as a means of increasing engagement and participation, such as those outlined in CSPAP, are required.

Another possible reason for our lack of significant findings may be due to the structure of the program, which consisted of fitness-based exercises (e.g., jumping jacks, squats, jogging on the spot, etc.). It is possible that the program was therefore less enjoyable than more game-like, or otherwise more engaging, activities. If students did not enjoy the sessions, they could have been less motivated to participate, and may not have been participating at an optimal aerobic intensity to elicit positive affective experiences associated with various changes in psychosocial outcomes [52]. A shift to more enjoyable DPA activities may therefore be worth exploring.

One type of physical activity that is gaining popularity for school DPA is the use of cognitively-engaging physical activity, or physically active academic lessons, which have been found to both increase activity levels and improve academic outcomes [53]. Specifically, cognitively engaging physical activity has been shown to positively influence aspects of executive functioning, brain-based processes known to enhance academic performance, to a greater extent than strictly fitness-based activities [54, 55]. Interestingly, research has also shown that higher levels of positive affect, following cognitively engaging physical activity, lead to (i.e., mediate) greater changes in executive functioning performance [54]. Thus, future research is encouraged to examine different types of classroom-based physical activity (i.e., cognitively engaging physical activity) that may lead to greater changes in positive affect and, ultimately, motivation for engaging in daily classroom-based physical activity sessions when compared to more fitness-based exercises that are currently prescribed (i.e., standard aerobic and resistance fitness activity).

### Limitations

There are a number of limitations to the current study. First, slightly less than half of eligible students consented to participate in the program evaluation. It is possible that those students who did not consent had lower levels of psychosocial well-being at baseline and thus, would have benefited the most from the program. Second, the unbalanced nature of our groups will have reduced statistical power; however, we did not have control over the poor response rate from the control group. Third, we did not measure baseline DPA practices prior to the onset of the intervention. Fourth, that we relied on teacher self-report for adherence to the program and changes in student behaviour post-intervention is also a limitation. However, that we found such low adherence is alarming given that recall or other biases (e.g., social desirability) may have resulted in teachers reporting higher than actual adherence. We also employed a rather lengthy survey over three-time points which may have resulted in response bias, social desirability, dissimulation or participant fatigue, any or all of which could have unintentionally contributed to our findings (see [56]). Finally, as this was a program evaluation, the study did not employ a randomized design. Strengths of the study include the relatively large sample, the inclusion of a control group, and the measurement of a broad range of child-reported psychosocial outcomes.

### Conclusions

At the outset of the paper, we noted the limitations of investigator-led interventions in school based settings. Seeking to evaluate a program designed and implemented outside the research context offered the chance to overcome some of the implementation challenges (e.g., sustainability and feasibility) noted in investigator-driven research. This approach is consistent with so-called effectiveness trials, where an intervention is evaluated in a real-world setting, typically without the controls normally in place for experimental research (i.e., efficacy trial; [57]). In this context, the program evaluated here did not improve psychosocial well-being of elementary school-aged children more than existing practices. However, overall adherence to the program was relatively poor and importantly, it did not appear to have a negative effect on the students. Future research should further explore the impact of DPA on psychosocial well-being in children and youth, in addition to how these outcomes may be mediated by other factors including, overall physical activity levels, physical fitness, academic achievement, and cognitions. Importantly, further work is needed on how best to support teachers and administrators in the implementation of DPA, including what structure and types of activities will provide optimal outcomes, in order to enhance the learning environment for elementary school-aged children and positively impact their overall development.

## Abbreviations

DPA: Daily physical activity
MVPA: Moderate to vigorous physical activity
PAQ-C: Physical Activity Questionnaire for Older Children
SDQ: Strengths and Difficulties Questionnaire
